# Determination of Phenolics and Flavonoids of Some Useful Medicinal Plants and Bioassay-Guided Fractionation Substances of *Sclerocarya birrea* (A. Rich) Hochst Stem (Bark) Extract and Their Efficacy Against *Salmonella typhi*

**DOI:** 10.3389/fchem.2021.670530

**Published:** 2021-07-27

**Authors:** Muhammad Salihu Abdallah, Muskhazli Mustafa, Meenakshii A/P. Nallappan, Sangho Choi, Jin-Hyub Paik, Go Rusea

**Affiliations:** ^1^ Department of Biology, Universiti Putra Malaysia, Serdang, Malaysia; ^2^ Department of Microbiology, Yobe State University, Damaturu, Nigeria; ^3^ Korea Research Institute of Bioscience and Biotechnology (KRIBB), Daejeon, South Korea

**Keywords:** Anacardiaceae, antibacterial, catechin, Gallic acid, herbs, solvent partitioning, LCHR-MS

## Abstract

Gallic acid and catechin are the most abundant phenolic and flavonoid contents found in all plant extracts. The contents and the bioassay-guided fractionating substances of the *Sclerocarya birrea* (A. Rich) Hochst (Anacardiaceae) fraction played vital roles. The goals of the study were to determine the contents of some useful medicinal plants and the bioassay-guided fractionation substances of *S. birrea* fraction compounds capable of acting against *Salmonella* isolate using LC-MS/LC-HRMS (Dionex ultimate 3000 RS UPLC with Thermo Scientific Q Exactive Orbitrap Hybrid Tandem Mass Spectrometer). The Folin–Ciocalteu reagent procedure and flavonoid content determination were conducted spectrophotometrically. Bioassay-guided fractionation, chronological partitioning, and screening of the antibacterial action against *Salmonella typhi* were performed. The ethyl acetate fraction extracts of *S. birrea* stem (bark) extract were analyzed using LC-MS/LC-HRMS. The gallic acid content increased tremendously in *Vachellia nilotica* (L.) P.J.H. Hurter and Mabb (Fabaceae) pod extracts with curve fitting (*R* ^2^ = 0.9958). Catechin content increase was significantly increased in *S. birrea* stem (bark) extracts followed by that of *V. nilotica* pod extracts with curve fitting (*R* ^2^ = 0.9993); they were all significantly different in the *Guiera senegalensis* J.F. Gmel. and the *Leptadenia lanceolata* (Poir.) Goyder leaves extracts at *p* value <0.0001. Subsequently, 10 mg/ml of *S. birrea* stem (bark) ethyl acetate fraction extract was the MIC, where no MBC was recorded and susceptible to the positive control with the highest inhibition zone, followed by the ethyl acetate fraction extract at 10 mg/ml (9.7 ± 0.0) at Turkey’s *p* < 0.0001. Vidarabine is one of the novel compounds, specifically having antimicrobial actions, found in the *S. birrea* stem (bark). Reasonable amounts of phenolic and flavonoid contents determined the actions of the individual plant extract.

## Introduction

Plant-derived medicines are widely used because they are relatively safer than the orthodox ones. Antibiotic resistance has become a global concern and we are threatened by the emergence of multidrug resistance pathogens (Adoum, 2106). The use of medicinal plant extracts to treat infectious diseases is an age-old practice that relies on traditional medicine (Paudel et al., 2018). Phytochemical ingredients in various parts of the plant vary significantly (Adoum, 2106). The *V. nilotica* pod extracts have been used in folk medicine (Mustafa et al., 1999). Subsequently, methanolic extracts were revealed to have higher total phenolic contents and good antioxidant activities compared to other extracts. They possessed very good bioactive ingredients for counteracting various ailments (Johari and Khong, 2019).

For *G. senegalensis,* leaves were taken for many purposes like; pulmonary and respiratory problems, colic and diarrhea, syphilis, beriberi, leprosy, rheumatism, diuresis, impotence and expurgation (Anka et al., 2020). Many bioactive compounds were identified from *G. senegalensis,* including antioxidants, high contents of polyphenols, tannins, flavonoids, glycosides, and essential oils (Gurama et al., 2020). Subsequently, *L. lanceolata* has been used locally in treating many ailments, including some physiological imbalances like hypertension, sex ineffectiveness, and milk drying (Malgwi et al., 2019). Phytochemicals have clearly demonstrated a number of different compounds such as alkaloids, flavonoids, phenolic, glycosides, saponins, and tannins in a plant (Mustapa et al., 2018). Phenolics and flavonoids contents of the *L. lanceolata* were also regarded to be quantified and determined using HPLC as described by (Ja’afar et al., 2013).

Another native African tree is *S. birrea*, known locally as “Marula.” Its stem bark, roots, leaves, and fruits contained many chemical components such as polyphenols, tannins, coumarins, flavonoids, triterpenoids, and phytosterols. It is used as food and traditional medicine for their ailments (Mariod and Abdel). The bioassay-guided fractionation is a technique for plant extract profiling and screening of the bioactive compounds as potential sources of bio-based new drugs. The fractionation procedure is employed to screen natural products in the plant extracts better (Malviya, 2019). The bioassay-guided fractionation of methanolic extracts of *Eupatorium triplinerve* Vahl (Asteraceae) revealed active antimicrobial components in some solvents, notably hexane, dichloromethane, and ethyl acetate against *Escherichia coli* at 16 and 31 mg/ml and *Pseudomonas aeruginosa* at 31 and 62 mg/ml MICs (Regina et al., 2015). Subsequently, the *Salmonella paratyphi* is a Gram-negative, rod-shaped, facultative anaerobe, non-encapsulated, non-spore-forming, flagellated and motile bacterium. Three serotypes of *S. paratyphi* were described: *S. paratyphi* A, B, and C worldwide (Panezai et al., 2018). *S. paratyphi* transmission was through the fecal-oral route or via eating of unclean food/water and coming in contact with chronic asymptomatic carriers (Panezai et al., 2018). Gastrointestinal ailments are among the major health concerns in most African countries and in other undeveloped countries, which devastated the world by causing high morbidity and fatality rates (Manzo et al., 2017).

Lastly, most of the *S. birrea* secondary metabolites were also determined using liquid chromatography-mass spectroscopy and NMR. Notably, flavonoids were responsible for fighting various ailments (Mc et al., 2017). The present study is the first to explore selected medicinal plants’ contents from Yobe State, Nigeria. Vidarabine, a novel compound, was identified in the *S. birrea* stem (bark) fraction extract contrary to the earlier studies. The goals of this study were to determine the active compounds of some useful medicinal plants using LC-MS and the bioassay-guided fractionating substances from *S*. *birrea* stem (bark) fraction extract’s potency against bacterial isolates.

## Materials and Methods

### Collection of Plant Materials and Their Identifications

The plants voucher specimens collected were processed following a standard herbarium technique (Bridson and Forman, 1999). Subsequently, the samples were identified and authenticated by a plant taxonomist, Dr. Yusuf Nuhu, from the Department of Plant Biology, Bayero University Kano, Nigeria. All the identified plant specimens were given voucher numbers and deposited at the University Herbarium for reference purposes.

### Sample Preparation

The plant extracts (crude extracts of the leaf, stem (bark) and pods of *Guiera* senegalensis J (Combretaceae), *Leptadenia lanceolata* (Apocynaceae)*, S. birrea*, and *V. nilotica*) were individually dissolved in methanol and subjected to sonication for 1 h. Subsequently, after the sonication process, the extracts were kept overnight at 4°C. Then, the extracts were centrifuged at 5500 rpm for 10 min (10°C). Finally, the supernatants were used for analyses (Chan et al., 2014).

### Total Phenolic Contents

A 0.1 ML of the sample (standard) methanol was reacted with 0.5 ML of 10-fold diluted Folin–Ciocalteu reagent, followed by 0.4 ML of 7.5% (w/v) sodium bicarbonate solution. After incubation at 40°C for 30 min, 200 μg/ML of the reaction mixture was placed into a 96-well plate and the absorbance was recorded at 760 nm spectrophotometrically. Gallic acid was used as the standard (Chan et al., 2014).

### Total Flavonoid Contents

A 25 µL of sample (standard) methanol was mixed with 125 µL of ultrapure water and 7.5 µL of 5% (w/v) sodium nitrite solution. After 6 min, 15 µL of 2% (w/v, in ultrapure water) aluminum chloride solution was added and the mixture was allowed to stand for 5min. Then, 50 µL of 1 min sodium hydroxide and 27.5 µL of ultrapure water were serially added to the mixture and the mixture was vortexed for 10 s. Finally, 200 µL of the reaction mixture was placed into a 96-well plate and the absorbance of the mixture was measured at 510 nm (+); catechin was used as the standard (Ho et al., 2014).

### Preparation of the Ethanol Extract and the Fractions

A powdered form of *S. birrea* stem bark methanolic stem (bark) extract (20 g) was immersed in methanol for some hours, subsequently extracted using a standard separating funnel, and the solvent was evaporated under vacuum. The same extract was then suspended in water and partitioned successively with n-hexane, chloroform, and ethyl acetate. Each fraction was then evaporated in a vacuum with their respective yields. The extracts were concentrated under reduced pressure at 50°C and lyophilized to obtain powder for further use (Huang et al., 2011). Moreover, fractions of the solvents used were dissolved in DMSO in mg at variant concentrations for the antibacterial actions to affect their modes of action against the bacterial isolate (Huang et al., 2011; Cunha et al., 2017).

### Solvent Partitioning

**FIGURE 1:**
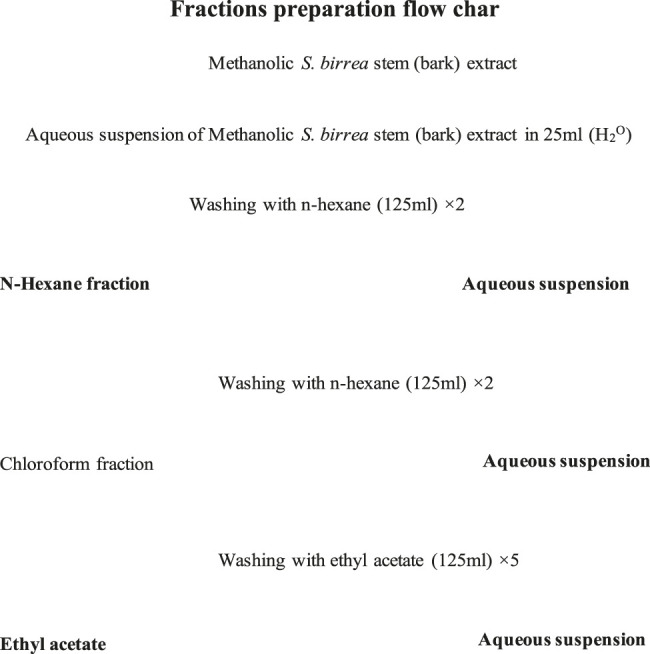
Liquid–Liquid partitions. Fraction preparation flowchart.

The solvent partitioning was done through a procedure used by (Jamil et al., 2012), with a few modifications. The crude plant extract was subjected to bioassay-guided fractionation with solubilization in water and a chronological partition with n-hexane (2 × 125 ml), chloroform (2 × 125 ml), and ethyl acetate (5 × 125 ml) as indicated in Figure 1. Then, each of the fractions obtained was evaporated to dryness and later screened by the antibacterial assay (Mc et al., 2017; Cunha et al., 2017).

### Testing Isolate Using the Fraction Extracts

Broth culture of the isolates was adjusted to correspond to a McFarland standard (Thermo Scientific) (∼10^6^°Cfu/ml). The inoculum was streaked using a swab stick onto the Mueller–Hinton agar (HimediaR) and allowed to stand on five prepared paper discs of plant extract concentrations using sterilized forceps. The inoculated Mueller–Hinton agar (HimediaR) plates were incubated at 37°C for 24 h. The plates’ zones of inhibition were read and measured to the nearest mm in diameter and recorded in three different trials (Nneka et al., 2016). Furthermore, both the MICs and the MBCs of the respective fraction extracts against the isolate (*S. typhi*) were tested following Abdullah et al. (2019) and Nneka et al. (2016), with a few modifications.

### LC-MS Procedure

The sample was analyzed using ultra-performance liquid chromatography with high-resolution mass spectrometry (LC-MS/LC-HRM) for the identification of compounds. The method used employed reversed-phase chromatography with a gradient range of solvent strengths. The online high-resolution accurate mass (HRAM) fragmentation library contains highly curated MS/MS and MSn spectra from different collision types and collision energies. Cloud Search was integrated into the compound discoverer along with other tools, such as predicted compositions based on high-resolution full MS and ChemSpider search, that helped partially identify the compounds. Built-in FISH scoring was used to verify hits from ChemSpider against the MS2 data.

#### Sample Preparation

One mg of extract was diluted in 1 ml methanol as the master stock (MS) and 10 μg/mL as the working stock (WS) in methanol before analysis. All samples were filtered with a 0.22 um PTFE membrane filter and transferred to 2 ml vials for analysis.

#### Operating Conditions

The results of the (LC-MS/LC-HRMS) were subjected to the Thermo Scientific Compound Discoverer software version 3.1 for online compound database matching using cloud and ChemSpider.

### Statistical Analyses

All experiments were carried out in triplicate and the results were expressed as mean values and standard deviations. One-way analysis of variance (ANOVA) was performed using GraphPad Prism version 8.0 and the differences between samples were compared using Turkey’s test (*p* < 0.05).

## Results

### Quantification of Phenolic Contents of *Sclerocarya birrea, Vachellia nilotica, Guiera senegalensis,* and *Leptadenia lanceolata* Extracts

**FIGURE 2:**
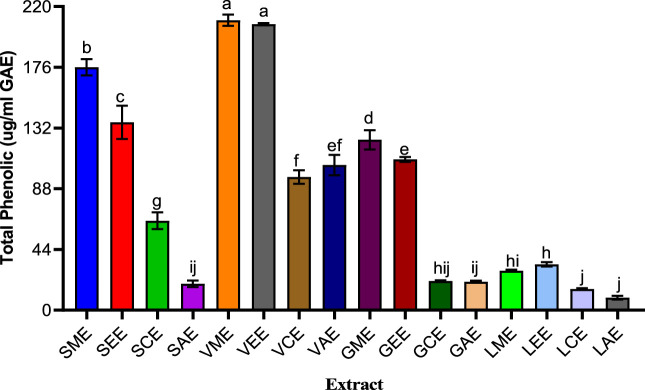
Various plants’ total phenolic contents. SME = *Sclerocarya birrea* methanolic extract; SEE = *Sclerocarya birrea* ethanolic extract; SCE = *Sclerocarya birrea* chloroform extract; SAE = *Sclerocarya birrea* water extract; VME = *Vachellia nilotica* methanolic extract; VEE = *Vachellia nilotica* ethanolic extract; VCE = *Vachellia nilotica* chloroform extract; VAE = *Vachellia nilotica* water extract; GME = *Gueira senegalensis* methanolic extract; GEE = *Gueira senegalensis* ethanolic extract; GCE = *Gueira senegalensis* chloroform extract; GAE = *Gueira senegalensis* water extract; LME = *Leptadenia lanceolata* methanolic extract; LEE = *Leptadenia lanceolata* ethanolic extract; LCE = *Leptadenia lanceolata* chloroform extract; LAE = *Leptadenia lanceolata* water extract. Letters a–j denote the levels of significance from one parameter to another. Bars of similar letters are not significantly different according to Turkey at *p* value < 0.0001.

Gallic acid increased tremendously, especially in *V. nilotica* pods extracts (210.063%) and *S. birrea* stem (bark) methanolic and ethanolic extracts with curve fitting (R2 = 0.9958), as shown in Figure 2, showing a very good curve fit across the medicinal plants, and all were significantly different against the *G. senegalensis* leaf extracts and the *L. lanceolata* leaves extracts with a very low content at F (15, 32) = 6,676, at *p* value < 0.0001.

### Quantification of Flavonoid Contents of *Sclerocarya birrea, Vachellia nilotica, Guiera senegalensis,* and *Leptadenia lanceolata* Extracts

Catechin was significantly increased was noticed in *S. birrea* stem (bark) extracts (38.81%), followed closely by *V. nilotica* pod extracts (25.6469%) with curve fitting (*R* ^2^ = 0.9993). The *S. birrea* stem (bark) extracts (38.81%) were followed closely by *V. nilotica* pod flavonoid contents. The *S. birrea* stem (bark) methanolic and ethanolic extracts were the highest extracts with more flavonoid contents, followed by *V. nilotica* ethanolic pod extract and methanolic pod extract, which were also significantly different at *p* value < 0.0001 across the remaining extracts, which were revealed to have comparatively low contents, especially *L. lanceolata* leaf extracts.

### Bioassay-Guided Fractionating Substances of *Sclerocarya birrea* (A. Rich) Hochst. Stem (Bark) Extract Against *Salmonella typhi*

**FIGURE 3:**
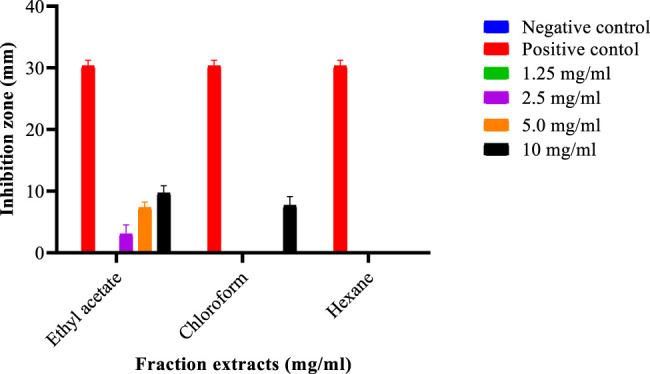
Inhibition zones of different *Sclerocarya birrea* stem (bark) fraction extracts against *Salmonella typhi.*

A 2.16 g *S. birrea* ethanolic stem (bark) crude extract had procured a 10.8% yield. The same extract yielded the following: 0.165 g residue of n-hexane with a fraction percentage yield of 2.05%; 0.0133 g chloroform with a fraction percentage yield of 1.65%; 0.2645 g ethyl acetate with a fraction percentage yield of 32.88%. The final recovered yield was 0.4711 g, with the final % yield recovered being 58.5654, after partitioning as depicted in Figure 1. Moreover, the fractions were subjected to antibacterial actions to affect their modes of action against the isolate. Subsequently, 2.5 mg/ml, 5 mg/ml, and 10 mg/ml happened to be the concentrations with such activities. These were subjected to the MICs, where 10 mg/ml of the *S. birrea* stem (bark) ethyl acetate fraction extract was the MIC and also there was no MBC on the fraction extract. Therefore, it inhibited the growth of the *S. typhi,* not killing it. Moreover, the statistical test showed that the isolate was susceptible to the positive control (ciprofloxacin 30.33 ± 0.0) as the highest inhibition zone, followed by the ethyl acetate fraction extract at 10 mg/ml (9.7 ± 0.0), and resistance was very significant to the respective concentrations on the remaining fraction extracts including the chloroform fraction extract at Turkey’s *p* < 0.0001 , as shown in Figure 3.

### LC-MS Analysis of Ethyl Acetate Fraction of *S. birrea* Stem (Bark) Extract Identified by MZCloud and ChemSpider

**FIGURE 4:**
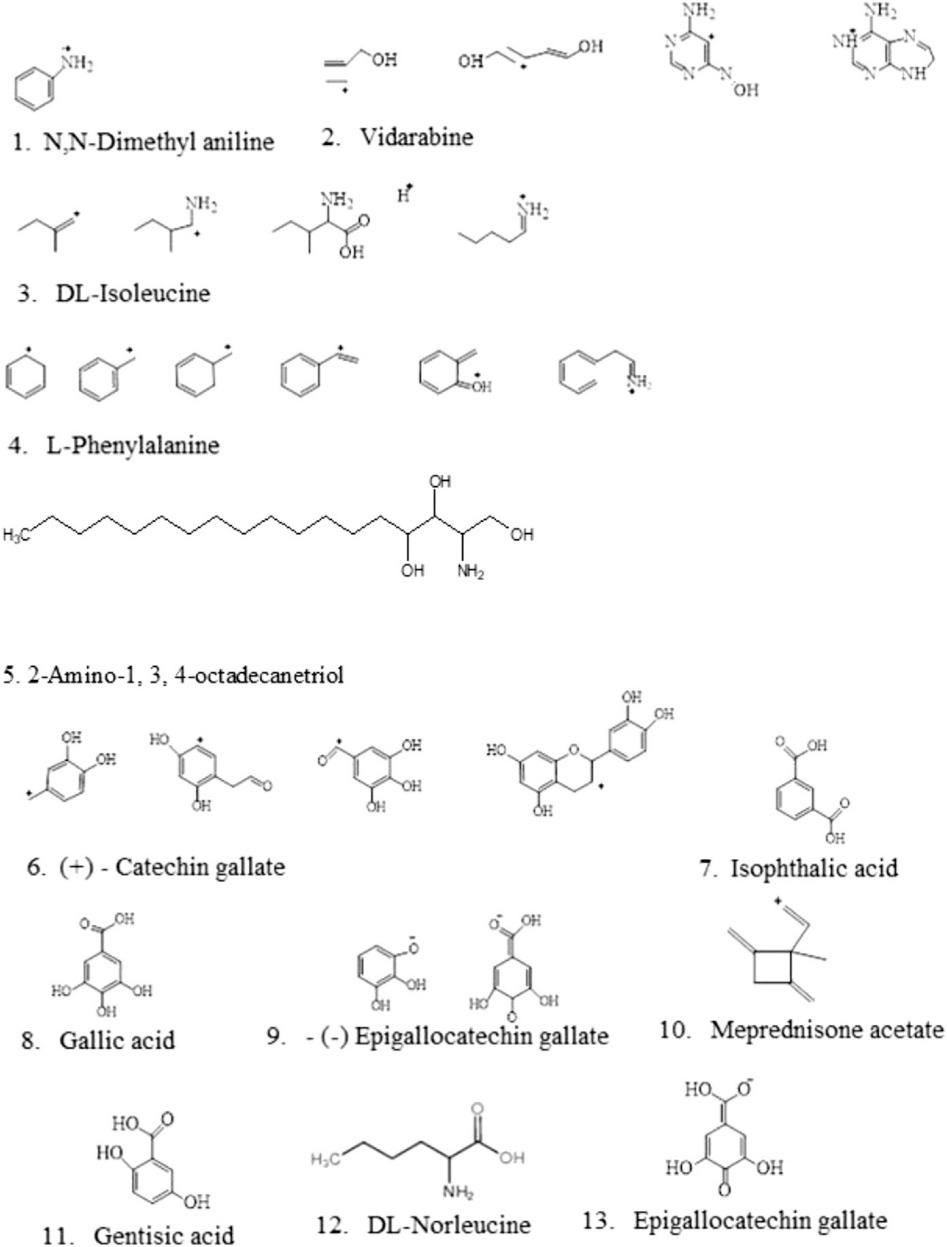
Identified compounds from *Sclerocarya birrea* ethyl acetate fraction extracts using LC-MS/LC-HRMS.

LC-HRMS identified 16 compounds with different fragmentation ions and various structures as far compounds. Vidarabine appeared to be the best compound with 99.9 MZCloud best full matches, followed by N, N-dimethylaniline, l-phenylalanine, catechin gallate, gallic acid, and meprednisone acetate as the least with partial matches, as shown in Table 1 and their respective structures in Figure 4. They showed the plant to be active against *S. typhi*. Subsequently, N, N-dimethyl aniline was identified by both MZCloud and ChemSpider (Table 2), with fully matched similar fragmentation rates, having an m/z of 122.0964 and a retention time of 0.043. Vidarabine was only identified by MZCloud with its fully and best matched fragmentation rate of 99.99 with m/z of 268.1049 and retention time of 0.148. DL-Isoleucine was identified by both MZCloud and ChemSpider with full matches of m/z of 132.1019 and a retention time of 1.296. DL-Norleucine showed different fragmentation rates using MZCloud and ChemSpider with the full matching of mz of 86.0964 with a retention time of 1.601.

**TABLE 1:** LC-MS/LC-HRMS analysis of the ethyl acetate fraction of *Sclerocarya birrea* stem (bark) extract identified by MZCloud and ChemSpider.

No	Name	Formula	Molecular weight	Main fragment ions MS^2^ (m/z)	Retention time (RT)	Area (max)
1.	N, N-Dimethyl aniline	C_8_ H_11_ N	121.0896	107.0730	0.043	1.30E ± 07
				122.0964		
2.	Vidarabine	C_10_ H_13_ N_5_ O_4_	267.0971	85.0290	0.148	5.22E ± 07
				136.0620		
				137.0458		
				268.1,049		
3.	N, N-Dimethyl aniline	C_8_ H_11_ N	121.0895	107.0730	0.386	5.16E ± 07
				122.0964		
4.	N, N-Dimethyl aniline	C_8_ H_11_ N	121.0896	107.0730	0.493	2.08E ± 08
				122.0964		
5.	DL-Isoleucine	C_6_ H_13_ N O_2_	131.0949	69.0699	1.296	7.04E ± 07
				86.0964		
				132.1019		
6.	DL-Norleucine	C_6_ H_13_ N O_2_	131.0950	86.0964	1.601	6.00E ± 07
7.	l-Phenylalanine	C_9_ H_11_ N O_2_	165.0793	103.0542	1.937	2.80E ± 07
				107.0491		
				120.0808		
				166.0863		
8.	Diisopropylethylamine	C_8_ H_19_ N	129.1521	130.1590	2.046	5.58E ± 07
9.	2-Amino-1,3,4-octadecanetriol	C_18_ H_39_ N O_3_	317.2935	318.3003	2.305	2.94E ± 07
10.	N-Methyl-4-piperidone	C_6_ H_11_ N O	113.0845	96.0813	2.371	3.48E ± 06
				114.0917		
11.	2-Amino-1,3,4-octadecanetriol	C_18_ H_39_ N O_3_	317.2935	256.2634	2.856	2.45E ± 07
				318.3005		
12.	N-Methyl-4-piperidone	C_6_ H_11_ N O	113.0845	73.0814	3.005	2.31E ± 07
				86.0970		
				96.0814		
				114.0918		
13.	N- Methyl-4-piperidone	C_6_ H_11_ N O	113.0845	96.0814	3.175	9.02E ± 06
				114.0918		
14.	2-Amino-1,3,4-octadecanetriol	C_18_ H_39_ N O_3_	317.2933	256.2435318.3003	11.240	1.62E ± 07
15.	(+)-Catechin gallate	C_22_ H_18_ O_10_	442.0886	123.0441	12.811	5.78E ± 06
				139.0390		
				151.0390		
				153.0182		
16.	1,8-Diazabicyclo [5.4.0]undec7-ene	C_9_ H_16_ N_2_	152.1312	96.0184	12.833	2.00E ± 07
				153.0586		
17.	2-Amino-1,3,4-octadecanetriol	C_18 _H_39_ NO_3_	317.2933	256.2435	13.440	2.42 ± 07
				300.2897		
				318.3003		
18.	2-Amino-1,3,4-octadecanetriol	C_18_ H_39_ NO_3_	317.2934	85.1012	13.618	4.41E ± 07
				256.2435		
				300.2897		
				318.3003		
19.	Gallic acid	C_7_ H_6_ O_5_	170.0210	69.0346	13.653	1.22E ± 07
				97.0295		
				125.0244		
				169.0143		
20.	Isophthalic acid	C_8_ H_6_ O_4_	166.0256	121.0295	13.698	1.07E ± 06
				165.0193		
21.	Gallic acid	C_7_ H_6_ O_5_	170.0205	69.0346	13.789	2.04E ± 07
				97.0295		
				125.0244		
				169.0143		
22.	Epigallocatechin gallate	C_22_ H_18_ O_11_	458.0842	125.0244	13.790	3.00E ± 06
				169.0143		
				165.0195		
				305.0667		
23.	Gallic acid	C_7_ H_6_ O_5_	170.0206	69.0346	14.100	1.34E ± 07
				97.0295		
				125.0244		
				169.0143		
24.	2-Amino-1,3,4-octadecanetriol	C_18_ H_39_ NO_3_	317.2910	256.2435	14.118	1.14E ± 07
				300.2897		
				318.3003		
25.	1,8-Diazabicyclo [5.4.0]undec-7-ene	C_9_ H_16_ N_2_	152.1315	96.0807	14.367	7.41E ± 06
				153.1386		
26.	Gallic acid	C_7_ H_6_ O_5_	170.0206	69.0346	15.268	1.25E ± 07
				97.0295		
				125.0244		
				169.0143		
27.	Meprednisone acetate	C_24_ H_30_ O_6_	414.2042	119.0858	15.444	1.34E ± 06
28.	Gallic acid	C_7_ H_6_ O_5_	170.0210	69.0346	15.504	2.63E ± 07
				97.0295		
				125.0244		
				169.0143		
29.	Gallic acid	C_7_ H_6_ O_5_	170.0207	69.0346	15.657	1.92E ± 07
				97.0295		
				125.0244		
				169.0143		
30.	Gentisic acid	C_7_ H_6_ O_4_	154.0260	109.0295	15.734	1.33E ± 06
				153.0193		
31.	Gallic acid	C_7_ H_6_ O_5_	170.0210	69.0346	15.735	1.25E ± 07
				97.0295		
				125.0244		
				169.0143		
32.	2-Amino-1,3,4-octadecanetriol	C_18_ H_39_ NO_3_	317.2926	85.1012	15.741	1.73E ± 08
				256.2435		
				300.2897		
				318.3003		
33.	Gallic acid	C_7_ H_6_ O_5_	170.0210	69.0346	15.879	1.47E ± 07
				97.0295		
				125.0244		
				169.0143		
34.	1,8-Diazabicyclo [5.4.0]undec-7-ene	C_9_ H_16_ N_2_	152.1315	153.1386	16.048	7.35E ± 06
35.	Epigallocatechin gallate	C_22_ H_18_ O_11_	458.0847	125.0244	16.221	1.76E ± 06
				169.0143		
36.	2-Amino-1,3,4-octadecanetriol	C_18_ H_39_ NO_3_	317.2927	85.1012	16.534	2.95E ± 07
				256.2435		
				300.2897		
				318.3003		
37.	2-Amino-1,3,4-octadecanetriol	C_18_ H_39_ NO_3_	317.2935	85.1012	19.581	3.01E ± 07
				256.2435		
				300.2897		
				318.3003		
38.	Gallic acid	C_7_ H_6_ O_5_	170.0206	69.0346	19.889	1.36E ± 06
				97.0295		
				125.0244		
				169.0143		

**TABLE 2:** 2.8.2 Operating conditions.

System	Dionex ultimate 3000 RS UPLC with Thermo Scientific Q Exactive Orbitrap Hybrid Tandem Mass Spectrometer	
Column	Waters XBridge TM C18, 2.5 um, 2.1 mm i.d x 50 mm	
Column temperature	40o^C^	
Mobile phase	A = 0.1% formic acid in water	
Separation gradient	Time (min)	%B
	0.0	10
	15.0	100
	20.0	100
Flow rate	0.4 ml/min	
Injection volume	5 uL	
m/z range	100–1,500	
Ion source and polarity	ESI (+ve and -ve)	
Resolution	70,000 (full MS scan) and 35,000 (ddMS2 scan)	
Collision energy	25, 40, and 55	
Software for data analysis	Compound discoverer 3.1	
Workflow description	Natural product unknown ID w stats online and local database searches (untargeted food research ID workflow with statistics: Detect and identify unknown compounds with differential analysis). Perform retention time alignment, unknown compound detection, and compound grouping across all samples. Predict elemental compositions for all compounds and hide chemical background (using blank samples). Identify compounds using MZCloud (ddMS2 and/or DIA), ChemSpider (exact mass or formula), and local database searches against mass lists (exact mass with or without RT) and mzVault spectral libraries. Perform spectral similarity search against MZCloud for compounds with ddMS2. Apply mzLogic to rank order structure candidates from ChemSpider and mass list matches. Apply spectral distance scoring to ChemSpider and mass list matches and perform differential analysis on detected compounds	

Moreover, l-phenylalanine was the second-best in having full matches with various fragmentation rates of 99.7, m/z (166.0863), and retention times [1.937]. Diisopropylethylamine showed full matches of fragmentations in only MZCloud despite being detected by both m/z (130.1590) and retention time [2.046]. 2-Amino-1,3,4-octadecanetriol revealed full matches with different fragmentation rates, m/z (318.3003), and retention time [2.305–19.581]. N-Methyl-4-piperidone had partial matches of fragmentation rates only revealed by ChemSpider along with the structures and m/z (114.0918) and retention time [2.371–3.175]. (+)-Catechin gallate had full matches of fragmentation rates revealed by ChemSpider and MZCloud and outstanding results with m/z (153.0182) and retention time [12.811]. 1,8-Diazabicyclo [5.4.0] undec7-ene also had full matches of fragmentation rates revealed by with its structures, m/z (318.3003), and retention time [13.440].

Consequently, gallic acid had different structures with full matches of fragmentation rates with little differences in both m/z (169.0143) and retention time [13.653–19.889]. Isophthalic acid fragmentation rates were revealed by both databases with only full matches in MZCloud, 165.0193 m/z with a retention time of 13.698. Epigallocatechin gallate’s full matches of different fragmentation rates were revealed by both databases with the respective structures, m/z (305.0667), and retention time [13.790–16.221]. Meprednisone acetate’s partial matches of the fragmentation rates were revealed by both databases, being the only compound with such attributes in this analysis and fraction with this structure, with 119.0858 m/z and retention time of 15.444. Lastly, gentisic acid fully matched fragmentations were revealed by MZCloud and merely by ChemSpider with few structures, 153.0193 m/z and retention time of 15.734, as shown in Table 1 and Figure 4.

Detailed information regarding the profiled compounds identified using LC-MS/LC-HRMS, with the aid of MZCloud and ChemSpider software, their respective chemical structures, molecular weights, fragmentation rates, and their retention time was extracted from the *S. birrea* stem (bark) extract as depicted.

## Discussion

Medicinal plants were regarded as valuable and most useful natural resources used for the invention of new novel drugs. Many compounds were better known as attributes to the efficacies of the used medicinal plants in treating many ailments caused by microbes (Hajara et al., 2018). The *S. birrea* fruit is rich in so many minerals and phenolic compounds (tannins, catechin, and hydroxycinnamic acid). Moreover, it is rich in polyphenols, tannins, flavonoids, alkaloids, saponins, coumarins, triterpenoids, phytosterols, and quercetin and also in many flavonoid derivatives. It has been reported that its stem bark shared similar constituents as those of both the leaves and fruits (Ojewole et al., 2009). Nevertheless, the methanolic extracts were revealed to have higher total phenolic contents and good antioxidant activities compared to the other extracts and possessed very good bioactive ingredients to counteract various ailments (Johari and Khong, 2019). In the present study, gallic acid increased tremendously, especially in *V. nilotica* pods extracts (210.063%) and *S. birrea* stem (bark) methanolic and ethanolic extracts with curve fitting (*R* ^2^ = 0.9958), as shown in Figure 2. This turned to be very good across the used medicinal plants. There were significant differences against the *G. senegalensis* leaf extracts and the *L. lanceolata* leaves extracts with very low content at F (15, 32) = 6,676, at *p* value < 0.0001.

**FIGURE 5:**
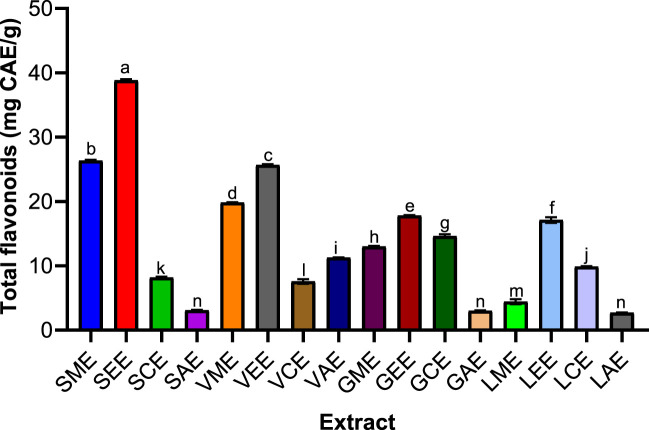
Various plants’ total flavonoid contents. SME = *Sclerocarya birrea* methanolic extract; SEE = *Sclerocarya birrea* ethanolic extract; SCE = *Sclerocarya birrea* chloroform extract; SAE = *Sclerocarya birrea* water extract; VME = *Vachellia nilotica* methanolic extract; VEE = *Vachellia nilotica* ethanolic extract; VCE = *Vachellia nilotica* chloroform extract; VAE = *Vachellia nilotica* water extract; GME = *Gueira senegalensis* methanolic extract; GEE = *Gueira senegalensis* ethanolic extract; GCE = *Gueira senegalensis* chloroform extract; GAE = *Gueira senegalensis* water extract; LME = *Leptadenia lanceolata* methanolic extract; LEE = *Leptadenia lanceolata* ethanolic extract; LCE = *Leptadenia lanceolata* chloroform extract; LAE = *Leptadenia lanceolata* water extract. Letters a–n denote the levels of significance from one parameter to another. Bars of similar letters are not significantly different according to Turkey at *p* value < 0.0001.

However, large catechin increases were noticed in *S. birrea* stem (bark) extracts (38.81%) followed closely by *V. nilotica* pod extracts (25.6469%) with curve fitting (*R* ^2^ = 0.9993), as shown in Figure 5. This happened to be an outstanding curve across the medicinal plants used in the study. The latter also agreed with the fact that *V. nilotica* pod extracts had been in used as a folk medicine due to their constituents (Mustafa et al., 1999; Johari and Khong, 2019). Similarly, secondary metabolites like tannins, flavonoids, saponins, steroids, terpenoids, and phenols were found to be rich in *S. birrea* stem bark extracts. Their therapeutic actions had been used to address many ailments in traditional alternative medicine like diarrhea (Manzo et al., 2017). Some phenolic compounds were also detected as parts of hydroxyl benzoic acids and cyclic polyol (Kura´nska et al., 2019).

Moreover, *G. senegalensis* leaves were used for many purposes, such as pulmonary and respiratory problems, colic and diarrhea, syphilis, beriberi, leprosy, rheumatism, diuresis, impotency, and expurgation (Anka et al., 2020). Many bioactive ingredients have been known to make the *G. senegalensis* active as antioxidants. It also had been reported to have high contents of polyphenols, tannins, flavonoids, glycosides, and essential oils (Gurama et al., 2020). This report contradicted the findings of the present study that *G. senegalensis* leaf extracts and *L. lanceolata* leaf extracts had very low contents at F (15, 32) = 6,676, at *p* value < 0.0001 with curve fitting (*R* ^2^ = 0.9993), as shown in Figure 4 (Ja’afar et al., 2013). *L. lanceolata* was quantified and determined to have reasonable contents (Mustapa et al., 2018) of phytochemicals such as alkaloids, flavonoids, phenolic, glycosides, saponins, and tannins, which contradicted the present findings.

Nevertheless, ethnopharmacology is a scientific discipline that is concerned with searching for substitute drugs in medicinal plants that have active ingredients to address health abnormalities. Hence, many medicinal plants’ properties were explored (Malgwi et al., 2019). The results of the various studies either confirmed or contradicted one another as stated by Malgwi et al., 2019; Khobe et al., 2017; SundayEne-OjoAtawodi et al., 2010. The methanolic extracts still indicated the presence of alkaloids, cardenolides, phlorotannins, steroids, tannins, and terpenoids (Malgwi et al., 2019). Nonetheless, polyphenols, guirenone, alkaloids, rhamnetin potentialities, as discovered using different techniques as described by (SundayEne-OjoAtawodi et al., 2010), were also present in *L. lanceolata*, despite their low contents, which differed from the present study. Subsequently, *S. birrea* stem (bark) methanolic extract, *S. birrea* stem (bark) ethanolic extract, *V. nilotica* ethanolic pods extract, and *V. nilotica* methanolic pod extracts happened to be the ones with reasonable phenolic contents, being significantly different from the rest of the plants extracts including their chloroform extracts. Notably, the least content was captured in *L. lanceolata* leaf extracts as depicted. Teke et al. (2011) have revealed that the n-hexane fraction appeared to have the least antibacterial action and the ethyl acetate fraction was the most active one against the *Staphylococcus aureus.* This was similar to the findings of the present study of 2.16 g *S. birrea* ethanolic stem (bark) crude extract with a procured yield of 10.8%. The same extract yielded residue of n-hexane of 0.165 g and a fraction percentage yield of 2.05%; 0.0133 g chloroform with a fraction percentage yield of 1.65%; 0.2645 g ethyl acetate with a fraction percentage yield of 32.88%. The final recovered yield was 0.4711 g, with the final % yield recovered being 58.5654, after partitioning as depicted in Figure 1. The same n-hexane fraction was the least followed by the chloroform fraction and again the ethyl acetate fraction extract of *S. birrea* stem (bark) was the active fraction against *S. typhi* isolate, as shown in Figure 3.

Subsequently, the 10 mg/ml of the *S. birrea* stem (bark) ethyl acetate fraction extract was the MIC and also no MBC on the fraction extract. It, therefore, inhibited the growth of the *S. typhi* but did not kill it, which was in accordance with the findings of Teke et al. (2011), where the antimicrobial actions were notably attributed to the presence of the identified compounds in both the crude extract and its fraction. Moreover, the work of Mushore & Matuvhunye (2013) found that ethyl acetate and the methanolic extracts had significant zones of inhibition close to that of the standard, and the activity of the water extract provided a basis for using water extracts in treating ailments traditionally and effectively. The latter agreed with some parts of the present study, whereas other parameters differed.

Nevertheless, the majority of the traditional herbal medications used in Africa are provided by practitioners who live within the populations. They trusted the system over time and are willing to assist patients with their knowledge and skills, sometimes at the least costs (Aliyu et al., 2015). Most of these herbal medicines are procured in their crude forms, although some pre-packaged pharmaceutical forms also exist. Evidently, interest in various traditional practices now exists among practitioners of modern medicine and there are a growing number of practitioners of traditional, indigenous, or alternative systems (Aliyu et al., 2015). Solvents play important roles in making extracts to be more potent against the test organisms. Ethyl acetate and methanolic extracts had significant zones of inhibition as stated by Mushore and Matuvhunye (2013), which conformed to the present study, with some distinctive evidence that the isolate was susceptible to the positive control (ciprofloxacin, 30.33 ± 0.0).

Moreover, bioassay-guided fractionation has been a technique for plant extract profiling of bioactive compounds for screening potential sources of new drugs of biological importance. The fractionation procedure, therefore, is employed for better screening of natural products in plant extracts (Malviya, 2019). It has been confirmed that the bioassay-guided fractionation of the methanolic extract of *E. triplinerve* revealed active antimicrobial components in some solvents, notably hexane, dichloromethane, and ethyl acetate against *Escherichia coli* at 16 and 31 mg/ml and against *Pseudomonas aeruginosa* 31 and 62 mg/ml MICs (Regina et al., 2015).

However, it is evident that most of the Enterobacteriaceae family contributed to the destruction of the epithelial tissues, which led to other abnormalities, not only gastroenteritis (Nakamoto et al., 2019). It had been proven that most *Salmonella* spp. causing gastroenteritis were foodborne, which indicated survival in warmer conditions, whereas viral gastroenteritis occurred in cooler conditions (Fletcher et al., 2015). Consequently, *S. birrea* stem bark, roots, leaves, and fruits contained a myriad of chemical components, which allowed people to use the plant as both a food and as a traditional medicinal plant for their ailments (Mariod and Abdelwahab, 2010).

Generally, bioactive ingredients like alkaloids, essential oils biterpenoids, and flavonoids were normally extracted via methanol and ethanol, which revealed sound actions against both Gram-positive and Gram-negative bacteria such as *S. aureus* (Mushore and Matuvhunye, 2013). Moreover, *S. birrea* has tremendous ethnotherapeutic attributes and pharmacological actions due to its bioactive ingredients, which act as medicines. The bioactive ingredients include polyphenols, tannins, coumarins, flavonoids, triterpenoids, and phytosterols. Its constituents have antidiarrheal, antidiabetic, anti-inflammatory, anti-microbial, antihypertensive, anticonvulsant, and anti-plasmodial effects (Ojewole et al., 2009). Its fruits are regarded as wild fruits for their uniqueness of nutritional values by containing ascorbic acid and other hydrocarbons. *S. birrea* seeds do possess some essential acids and oil, which also acted as ant-inflammatory, antidiabetic, analgesic, antiparasitic, antimicrobial, and antihypertensive agents (Mariod and Abdelwahab, 2010).

Importantly, plants derived antimicrobial agents are the best options for combating the increase of bacterial antibiotic resistance (Khameneh et al., 2019). Moreover, most of the *S. birrea* secondary metabolites were determined using liquid chromatography-mass spectroscopy and NMR, where notably, flavonoids were the majority fighting various ailments (Mc et al., 2017). As with the present study, (+)-catechin gallate with full matches of fragmentation rates was revealed with 153.0182 m/z and retention time of 12.811. Epigallocatechin gallate also had full matches of different fragmentation rates as revealed by both the respective structures, m/z (305.0667), and retention time [13.790–16.221]. These caused the plant extracts to be active and play important roles in curing many abnormal conditions such as chronic pains, depression, and Parkinson’s disease (Zeratsky, 2019).

Nevertheless, epigallocatechin gallate served as a valuable compound found mostly in green tea and synthetic food as probable additives. It has been documented to prevent different growths of both Gram-positive and Gram-negative bacteria capable of spoiling food since it possessed food antioxidant actions (Mahdi et al., 2019). It has been reported that gallic acid has many important effects, which include antioxidant and anti-inflammatory activities, as well as being able to address gastrointestinal, neuropsychological, and cardiovascular disorders (Kahkeshani et al., 2019). These findings were in line with our results that gallic acid possessed full matches of fragmentation rates with m/z (169.0143) and retention time [13.653–19.889], which signified the action of the extract on the test organisms. This was subsequently supported by the work that stated “epicatechin compound was identified with varying molecular weight and retention times (m/z 169.0145 [M-H- 289–272] and (m/z 425.0851 [M-H-304] fragmentation rates.” It was also in agreement with the yielding of catechin unit due to the loss of a gallic acid during fragmentation, as stated by Shoko et al. (2018). Subsequently, another compound was identified as 9, 10, 13-TriHOME with a peak of m/z 211.1227 [M-H-45–57] due to the loss of a carboxylic acid group and butyl and water fragments. Its secondary fragments were detected at m/z 171.0814 [M-H-71–87] and m/z 283.0403 [M-H-29–18] from the ethyl fragment. This yield was responsible for the inhibitory action of the fraction (Shoko et al., 2018).

The present study revealed that N, N-dimethyl had m/z 122.0964 and retention time of 0.043 with its full matches fragmentation rates in the fraction extract of *S. birrea.* These were similar to the findings of Skarpeli-Liati et al., 2012. Vidarabine was identified by MZCloud with its full and best matches fragmentation rates of 99.99 with m/z 268.1049 and retention time of 0.148 among the identified novel compounds in the study first found in the *S. birrea* stem (bark), which possessed antimicrobial attributes. This was supported by the work of Sunil et al. (2010), who reported that this compound played an important role, especially in inhibiting viral DNA. Hence, it served as an antiviral component. Moreover, DL-isoleucine, m/z (132.1019) and retention time [1.296], and DL-norleucine, mz (86.0964) with retention time [1.601], and their respective fragmentation rates were also present in reasonable amounts according to our findings. They were also found to be part of the novel compounds discovered in the sample. We attributed the plant action against the bacterial isolate to their presence. We also reported an amino acid that was non-polar and aided in protein synthesis, which was very crucial in humans, that was found in the diet after ingestion. It could also be found in other organisms like bacteria and had an effect on diabetes (Binns et al., 2016). Moreover, DL-norleucine proved to be active against Alzheimer’s disease (Binns et al., 2016). It was also revealed to be part of the novel compounds in this research. Indeed, no literature reported DL-norleucine to be part of *S. birrea* stem (bark) extract constituents.

Moreover, the present findings revealed that l-phenylalanine was the second-best in obtaining full matches with various fragmentation rates of 99.7, m/z (166.0863), and retention time [1.937] in the fraction extract, which conformed with the fact that l-phenylalanine is an essential amino acid-like other normal amino acids helping in the production of proteins and could also be obtained from food and other supplement sources like Marula. It is also regarded as aromatic and polar due to its benzyl chain (Zeratsky, 2019). It played an important role in curing many abnormal conditions such as chronic pains, depression, and Parkinson’s disease (Zeratsky, 2019). In addition, meprednisone acetate has partial matches of the fragmentation rates, being the only compound with such attribute in this analysis, which is also a new novel compound, as it was never discovered in the extract and the fraction used together with m/z 119.0858 in retention time of 15.444. Lastly, gentisic acid is also one of the newly identified compounds, having full matches of fragmentations revealed by MZCloud and merely by ChemSpider with few structures, m/z 153.0193 and retention time of 15.734. The **N-methyl-4-piperidone**, **1,8-diazabicyclo[5.4.0]undec-7-ene**, **2-amino-1,3,4-octadecanetriol**, **isophthalic acid,** and **diisopropylethylamine** are the remaining notable novel compounds identified in the *S. birrea* stem (bark) extract, as no literature revealed their presence, as shown in Table 1 and Figure 4.

## Conclusion

In the present research, the phenolic contents of the *V. nilotica* pod extracts (210.063%) and *S. birrea* stem (bark) and methanolic and ethanolic extracts with gallic acid curve (*R* ^2^ = 0.9958) were significantly different between the *G. senegalensis* and the *L. lanceolata* leaf extracts at *p* value < 0.0001. Likewise, the flavonoid contents showed similar trends as the phenolic contents were determined with catechin value (*R* ^2^ = 0.9993). Moreover, the reasonable amounts of phenolic and flavonoid contents determined the actions of the individual plant, notably toward the development of many valuable pharmaceutical products.

Moreover, the ethyl acetate fraction of the *S. birrea* stem bark extract happened to have the highest yield of the entire subfractionated solvent extracts and is the most potent against *S. typhi.* It only inhibited the growth but did not kill the test organism. Different compounds were identified with their structures as revealed by both MZCloud and ChemSpider using LC-MS/LC-HRMS. Vidarabine was the best compound with full matches of fragmentation rates although only MZCloud revealed its structure, followed by the essential amino acids and flavonoids such as catechin gallate and epigallocatechin and other phenolics like gallic acids and gentisic acids with all full matches of fragmentation rates across. Most importantly, meprednisone acetate appeared to be the least compound with partial matches of the fragmentation rates as revealed by both methods. Vidarabine happened to be among the novel compounds found in the *S. birrea* stem (bark), with outstanding results as compared to the remaining novel compounds such as N-methyl-4-piperidone, 1,8-diazabicyclo[5.4.0]undec-7-ene, 2-amino-1,3,4-octadecanetriol, or isophthalic acid. A reasonable amount of phenolic and flavonoid contents determined the actions of the individual plant.

Nevertheless, the listed compounds in Table 1 elucidated with the fact that some were unknown while some were known with different structural formulae, revealed different elution times, and retention times at different fragmentation rates. Moreover, some compound found in the *S. birrea* stem (bark) extract were first identified in this work. Lastly, such a plant needs to be studied further to see the effects of the various compounds found and their contents. Individual compounds would be further fully identified to perform an *in vivo* testing so as to ascertain and correlate with the present findings so far. Thus, the *S. birrea* stem (bark) fraction extract is an important plant product, which could lead to solutions to many human health problems.

## Data Availability

The original data presented in the study are included in the article/Supplementary Material; further inquiries can be directed to the corresponding author.
