# Supplementary material for: Determination of Phenolics and Flavonoids of Some Useful Medicinal Plants and Bioassay-Guided Fractionation Substances of Sclerocarya birrea (A. Rich) Hochst Stem (Bark) Extract and Their Efficacy Against Salmonella typhi
Source: Front Chem. 2021 Jul 27;9:670530. doi: 10.3389/fchem.2021.670530 (PMC8353516; doi:10.3389/fchem.2021.670530)
Supplement: Supplementary file 1 [file DataSheet1.docx]

| **No.** | **Name** | **Formula** | **Molecular Weight** | **Retention Time (RT)** |
| --- | --- | --- | --- | --- |
| 1. | N,N-Dimethylaniline | C_8_ H_11_ N | 121.0896 | 0.043 |

**mzCloud**

**ChemSpider**

| **No.** | **Name** | **Formula** | **Molecular Weight** | **Retention Time (RT)** |
| --- | --- | --- | --- | --- |
| 2. | Vidarabine | C_10_ H_13_ N_5_ O_4_ | 267.0971 | 0.148 |

**ChemSpider**

| **No.** | **Name** | **Formula** | **Molecular Weight** | **Retention Time (RT)** |
| --- | --- | --- | --- | --- |
| 3. | N,N-Dimethylaniline | C_8_ H_11_ N | 121.0895 | 0.386 |

**mzCloud**

**ChemSpider**

| **No.** | **Name** | **Formula** | **Molecular Weight** | **Retention Time (RT)** |
| --- | --- | --- | --- | --- |
| 4. | N,N-Dimethylaniline | C_8_ H_11_ N | 121.0896 | 0.493 |

**mzCloud**

**ChemSpider**

| **No.** | **Name** | **Formula** | **Molecular Weight** | **Retention Time (RT)** |
| --- | --- | --- | --- | --- |
| 5. | DL-Isoleucine | C_6_ H_13_ N O_2_ | 131.0949 | 1.296 |

**mzCloud**

**ChemSpider**

| **No.** | **Name** | **Formula** | **Molecular Weight** | **Retention Time (RT)** |
| --- | --- | --- | --- | --- |
| 6. | DL-Norleucine | C_6_ H_13_ N O_2_ | 131.0950 | 1.601 |

**mzCloud**

**ChemSpider**

| **No.** | **Name** | **Formula** | **Molecular Weight** | **Retention Time (RT)** |
| --- | --- | --- | --- | --- |
| 7. | L-Phenylalanine | C_9_ H_11_ N O_2_ | 165.0793 | 1.937 |

**mzCloud**

**ChemSpider**

| **No.** | **Name** | **Formula** | **Molecular Weight** | **Retention Time (RT)** |
| --- | --- | --- | --- | --- |
| 8. | Diisopropylethylamine | C_8_ H_19_ N | 129.1521 | 2.046 |

**mzCloud**

**ChemSpider**

| **No.** | **Name** | **Formula** | **Molecular Weight** | **Retention Time (RT)** |
| --- | --- | --- | --- | --- |
| 9. | 2-Amino-1,3,4-octadecanetriol | C_18_ H_39_ N O_3_ | 317.2935 | 2.305 |

**mzCloud**

**ChemSpider**

| **No.** | **Name** | **Formula** | **Molecular Weight** | **Retention Time (RT)** |
| --- | --- | --- | --- | --- |
| 10. | N-Methyl-4-piperidone | C_6_ H_11_ N O | 113.0845 | 2.371 |

**ChemSpider**

| **No.** | **Name** | **Formula** | **Molecular Weight** | **Retention Time (RT)** |
| --- | --- | --- | --- | --- |
| 11. | 2-Amino-1,3,4-octadecanetriol | C_18_ H_39_ N O_3_ | 317.2935 | 2.856 |

**mzCloud**

**ChemSpider**

| **No.** | **Name** | **Formula** | **Molecular Weight** | **Retention Time (RT)** |
| --- | --- | --- | --- | --- |
| 12. | N-Methyl-4-piperidone | C_6_ H_11_ N O | 113.0845 | 3.005 |

**ChemSpider**

| **No.** | **Name** | **Formula** | **Molecular Weight** | **Retention Time (RT)** |
| --- | --- | --- | --- | --- |
| 13. | N-Methyl-4-piperidone | C_6_ H_11_ N O | 113.0845 | 3.175 |

**ChemSpider**

| **No.** | **Name** | **Formula** | **Molecular Weight** | **Retention Time (RT)** |
| --- | --- | --- | --- | --- |
| 14. | 2-Amino-1,3,4-octadecanetriol | C_18_ H_39_ N O_3_ | 317.2933 | 11.240 |

**mzCloud**

**ChemSpider**

| **No.** | **Name** | **Formula** | **Molecular Weight** | **Retention Time (RT)** |
| --- | --- | --- | --- | --- |
| 15. | (+)-Catechin gallate | C_22_ H_18_ O_10_ | 442.0886 | 12.811 |

**mzCloud**

**ChemSpider**

| **No.** | **Name** | **Formula** | **Molecular Weight** | **Retention Time (RT)** |
| --- | --- | --- | --- | --- |
| 16. | 1,8-Diazabicyclo [5.4.0]undec-7-ene | C_9_ H_16_ N_2_ | 152.1312 | 12.833 |

**mzCloud**

**ChemSpider**

| **No.** | **Name** | **Formula** | **Molecular Weight** | **Retention Time (RT)** |
| --- | --- | --- | --- | --- |
| 17. | 2-Amino-1,3,4-octadecanetriol | C_18_ H_39_ N O_3_ | 317.2933 | 13.440 |

**mzCloud**

**ChemSpider**

| **No.** | **Name** | **Formula** | **Molecular Weight** | **Retention Time (RT)** |
| --- | --- | --- | --- | --- |
| 18. | 2-Amino-1,3,4-octadecanetriol | C_18_ H_39_ N O_3_ | 317.2934 | 13.618 |

**mzCloud**

**ChemSpider**

| **No.** | **Name** | **Formula** | **Molecular Weight** | **Retention Time (RT)** |
| --- | --- | --- | --- | --- |
| 19. | Gallic acid | C_7_ H_6_ O_5_ | 170.0210 | 13.653 |

**mzCloud**

**ChemSpider**

| **No.** | **Name** | **Formula** | **Molecular Weight** | **Retention Time (RT)** |
| --- | --- | --- | --- | --- |
| 20. | Isophthalic acid | C_8_ H_6_ O_4_ | 166.0256 | 13.698 |

**mzCloud**

| **No.** | **Name** | **Formula** | **Molecular Weight** | **Retention Time (RT)** |
| --- | --- | --- | --- | --- |
| 21. | Gallic acid | C_7_ H_6_ O_5_ | 170.0205 | 13.789 |

**mzCloud**

| **No.** | **Name** | **Formula** | **Molecular Weight** | **Retention Time (RT)** |
| --- | --- | --- | --- | --- |
| 22. | Epigallocatechin gallate | C_22_ H_18_ O_11_ | 458.0842 | 13.790 |

**mzCloud**

**ChemSpider**

| **No.** | **Name** | **Formula** | **Molecular Weight** | **Retention Time (RT)** |
| --- | --- | --- | --- | --- |
| 23. | Gallic acid | C_7_ H_6_ O_5_ | 170.0206 | 14.100 |

**mzCloud**

| **No.** | **Name** | **Formula** | **Molecular Weight** | **Retention Time (RT)** |
| --- | --- | --- | --- | --- |
| 24. | 2-Amino-1,3,4-octadecanetriol | C_18_ H_39_ N O_3_ | 317.2910 | 14.118 |

**mzCloud**

**ChemSpider**

| **No.** | **Name** | **Formula** | **Molecular Weight** | **Retention Time (RT)** |
| --- | --- | --- | --- | --- |
| 25. | 1,8-Diazabicyclo [5.4.0]undec-7-ene | C_9_ H_16_ N_2_ | 152.1315 | 14.367 |

**mzCloud**

**ChemSpider**

| **No.** | **Name** | **Formula** | **Molecular Weight** | **Retention Time (RT)** |
| --- | --- | --- | --- | --- |
| 26. | Gallic acid | C_7_ H_6_ O_5_ | 170.0206 | 15.268 |

**mzCloud**

| **No.** | **Name** | **Formula** | **Molecular Weight** | **Retention Time (RT)** |
| --- | --- | --- | --- | --- |
| 27. | Meprednisone Acetate | C_24_ H_30_ O_6_ | 414.2042 | 15.444 |

**ChemSpider**

| **No.** | **Name** | **Formula** | **Molecular Weight** | **Retention Time (RT)** |
| --- | --- | --- | --- | --- |
| 28 | Gallic acid | C_7_ H_6_ O_5_ | 170.0210 | 15.504 |

**mzCloud**

**ChemSpider**

| **No.** | **Name** | **Formula** | **Molecular Weight** | **Retention Time (RT)** |
| --- | --- | --- | --- | --- |
| 29 | Gallic acid | C_7_ H_6_ O_5_ | 170.0207 | 15.657 |

**mzCloud**

**ChemSpider**

| **No.** | **Name** | **Formula** | **Molecular Weight** | **Retention Time (RT)** |
| --- | --- | --- | --- | --- |
| 30 | Gentisic acid | C_7_ H_6_ O_4_ | 154.0260 | 15.734 |

**mzCloud**

**ChemSpider**

| **No.** | **Name** | **Formula** | **Molecular Weight** | **Retention Time (RT)** |
| --- | --- | --- | --- | --- |
| 31 | Gallic acid | C_7_ H_6_ O_5_ | 170.0210 | 15.735 |

**mzCloud**

**ChemSpider**

| **No.** | **Name** | **Formula** | **Molecular Weight** | **Retention Time (RT)** |
| --- | --- | --- | --- | --- |
| 32 | 2-Amino-1,3,4-octadecanetriol | C_18_ H_39_ N O_3_ | 317.2926 | 15.741 |

**mzCloud**

**ChemSpider**

| **No.** | **Name** | **Formula** | **Molecular Weight** | **Retention Time (RT)** |
| --- | --- | --- | --- | --- |
| 33 | Gallic acid | C_7_ H_6_ O_5_ | 170.0210 | 15.879 |

**mzCloud**

**ChemSpider**

| **No.** | **Name** | **Formula** | **Molecular Weight** | **Retention Time (RT)** |
| --- | --- | --- | --- | --- |
| 34 | 1,8-Diazabicyclo [5.4.0]undec-7-ene | C_9_ H_16_ N_2_ | 152.1315 | 16.048 |

**mzCloud**

**ChemSpider**

| **No.** | **Name** | **Formula** | **Molecular Weight** | **Retention Time (RT)** |
| --- | --- | --- | --- | --- |
| 35 | Epigallocatechin gallate | C_22_ H_18_ O_11_ | 458.0847 | 16.221 |

**mzCloud**

| **No.** | **Name** | **Formula** | **Molecular Weight** | **Retention Time (RT)** |
| --- | --- | --- | --- | --- |
| 36 | 2-Amino-1,3,4-octadecanetriol | C_18_ H_39_ N O_3_ | 317.2927 | 16.534 |

**mzCloud**

**ChemSpider**

| **No.** | **Name** | **Formula** | **Molecular Weight** | **Retention Time (RT)** |
| --- | --- | --- | --- | --- |
| 37 | 2-Amino-1,3,4-octadecanetriol | C_18_ H_39_ N O_3_ | 317.2935 | 19.581 |

**mzCloud**

**ChemSpider**

| **No.** | **Name** | **Formula** | **Molecular Weight** | **Retention Time (RT)** |
| --- | --- | --- | --- | --- |
| 38 | Gallic acid | C_7_ H_6_ O_5_ | 170.0206 | 19.889 |

**mzCloud**
